# Comparative Analysis of Redox Homeostasis Biomarkers in Patients with Psoriasis and Atopic Dermatitis

**DOI:** 10.3390/antiox12101875

**Published:** 2023-10-18

**Authors:** Aleksandra Klisic, Mirjana Bakic, Vesna Karanikolic

**Affiliations:** 1 Faculty of Medicine, University of Montenegro, 81000 Podgorica, Montenegro; 2 Center for Laboratory Diagnostics, Primary Health Care Center, 81000 Podgorica, Montenegro; 3 Clinic for Dermatovenerology, Clinical Center of Montenegro, 81000 Podgorica, Montenegro; 4 Clinic for Skin Diseases of the Clinical Center Nis, School of Medicine, University of Nis, 18000 Nis, Serbia

**Keywords:** atopic dermatitis, inflammation, oxidative stress, psoriasis

## Abstract

**Aim:** There are no studies regarding comparative analysis of serum biomarkers of oxidative stress in patients with psoriasis (PsO) and atopic dermatitis (AD). We aimed to compare the serum redox homeostasis parameters in patients with PsO vs. AD in an attempt to find the sensitive and specific oxidative stress biomarker that could best reflect the existence of one of these disease entities. **Methods:** Forty patients with PsO and forty patients with AD were consecutively included in this cross-sectional study. Parameters of redox homeostasis, i.e., pro-oxidants [malondialdehyde (MDA) and advanced oxidation protein products (AOPP)] and antioxidants [catalase (CAT) and superoxide dismutase (SOD)] were determined. **Results:** There was no difference in oxidative stress biomarkers between the PsO and AD group, except for higher CAT activity in the AD group (*p* < 0.001). Among all examined redox homeostasis biomarkers, ROC analysis showed that only CAT exhibited good diagnostic accuracy (AUC = 0.719) in the discrimination of patients with PsO vs. AD, with 0.436 U/L as the cut-off value of CAT activity. **Conclusions:** The CAT exhibited good diagnostic accuracy in the discrimination of patients with AD from those with PsO. The obtained results could suggest the importance of the use of antioxidants as a potential therapeutic strategy in the treatment of these two skin inflammatory diseases.

## 1. Introduction

Psoriasis (PsO) and atopic dermatitis (AD) are chronic diseases closely related to inflammation and oxidative stress [1]. Both diseases affect not only the skin but multiple organs due to their systemic nature [2].

The prevalence of AD varies from 10 to 30% in youngsters to up to 10% in the adult population. The prevalence of PsO in children is nearly 1.4%, whereas it ranges between 0.5 to 11.5% in adults [2,3].

The Th1-/Th17 immune response is assumed to be the key initiator of PsO development, whereas the Th2 immune response seems to be the major mechanism that triggers AD [2,4]. Despite these differences and despite the fact that the clinical manifestations of AD are being manifested in early childhood [5], these two disease entities have some similarities related to the increase in proinflammatory cytokines and enhanced pro-oxidant milieu [1,6,7,8], which lead to the destruction of proteins, DNA, and lipids by free radicals [7]. Namely, the abnormal epidermis differentiation/hyperproliferation is triggered by increased cytokines production (i.e., interleukin (IL)-12/23, IL-22, IL-17, tumor necrosis factor-alpha (TNF-α), IL-6, IL-4, IL-13, etc.)) that favor free radicals production, leading to diminishing already compromised non-enzymatic and enzymatic antioxidant system defense and creating a vicious circle between inflammation and redox imbalance in PsO and AD pathogenesis [1,9].

There is a variety of treatment options for PsO and AD [2,5]. Topical corticosteroids, retinoids, and vitamin D analogs are applied for mild PsO. In addition to phototherapy, systemic agents (e.g., methotrexate, acitretin, and cyclosporine) are used for severe forms of the disease [5]. Similarly, topical corticosteroids are the first-line treatment option in patients with AD. The second/third-line treatment options are the medications that disable T and/or B cell activation (e.g., methotrexate, cyclosporine, azathioprine) [5]. Biologic therapy is monoclonal antibodies (mAbs) that target major pathways of cytokines [2]. Given the fact that PsO is related to the TNFα-IL23-IL17 axis modulation, which has a major impact on inflammatory cascade, biologic therapy has emerged as the promising treatment for targeting mentioned cytokines in recent decades, although it has been much more expensive than the conventional treatment options [5]. TNF inhibitors showed satisfactory results in patients with PsO, which is not the case in AD patients [2]. 

A lot of effort has been made to identify biomarkers that would best reflect the disease onset and progression [10]. However, a reliable marker that would differentiate PsO from AD has rarely been examined [11,12] and has not been established yet. Additionally, there are no studies regarding comparative analysis of serum biomarkers of oxidative stress in these disease entities. A few previous studies [11,12] examined biomarkers of oxidative stress in urine. Shimamoto et al. [11] evaluated urinary 8-hydroxy-2’ -deoxyguanosine (8-OHdG) (i.e., a biomarker of oxidative damage of DNA) and did not find the difference between PsO (n = 25) and AD (n = 40), although the levels of this biomarker in urine were higher in PsO and AD in comparison with healthy controls, suggesting the role of oxidative stress in both diseases.

Concerning the previously mentioned, we aimed to compare the serum redox homeostasis parameters in patients with PsO and AD and to detect the sensitive and specific oxidative stress biomarker that could best reflect the existence of one of these disease entities.

## 2. Materials and Methods

### 2.1. Patients

The current study was derived from the recently conducted study that examined some inflammation and hematological biomarkers in patients with PsO and AD [13]. A total of 40 patients with PsO and 40 patients with AD were consecutively included in this cross-sectional study in a period between February and May 2023. All participants were examined by a dermatologist and screened for inclusion in the study. The research was carried out following the Helsinki Medical Declaration ethical principles. Each patient signed an informed consent. The protocol of the study was approved by the Institutional Ethics Committee. 

The inclusion criteria were the cases with a diagnosis of PsO or AD who accepted voluntarily to be included in the study, as described previously [13]. Patients who used biologic therapy, antioxidant supplements during the last three months, pregnant women, patients with malignancies, mental disorders, stroke, non-plaque types of PsO (i.e., pustular, erythrodermic, guttate), skin diseases, and autoimmune diseases other than PsO and AD were excluded from the examination. 

Each patient filled in the questionnaire related to medication use, demographic data, and lifestyle habits.

For quantitative measurement of the intensity of pruritus, a numerical rating scale was used. It ranges from 0 to 10 (where 0 was the lowest intensity and 10 was the pruritus of the highest intensity).

### 2.2. Methods 

The anthropometric measurements (i.e., body height, weight, and BMI) and blood sampling were conducted the same morning after at least 8 h of fasting state. 

After sampling in serum separator and clot activator tubes, the samples were left to clot for about 30 min and then were centrifuged. The sera samples were used for the following biochemical analyses: metabolic parameters, i.e., triglycerides (TG), total cholesterol (TC), high-density lipoprotein cholesterol (HDL-c), low-density lipoprotein cholesterol (LDL-c) and glucose were measured on Roche Cobas c501 chemistry analyzer (Roche Diagnostics GmbH, Mannheim, Germany); parameters of redox homeostasis were determined spectrophotometrically. Malondialdehyde (MDA), as a lipid peroxidation biomarker, was related to the measurement of thiobarbituric acid reactive substances using the test with thiobarbituric acid [14,15]. Advanced oxidation protein products (AOPP), as indicators of the oxidative damage of proteins, were analyzed following the recommendations of the Witko-Sarsat method, related to reaction with glacial acetic acid and potassium iodide [16,17]. The activity of catalase (CAT), as an enzymatic antioxidant, was determined by the release of oxygen from hydrogen peroxide (H_2_O_2_) following the formation of the ammonium molybdate-stable complex [18]. The activity of superoxide dismutase (SOD) was measured using Misra and Fridovich method [19].

### 2.3. Statistical Analysis

Statistical analysis was performed using the SPSS statistical package (version 18.0 for Windows, SPSS, Chicago, IL, USA). The distribution of variables was tested with the Shapiro–Wilk test.

Data are presented as counts and percentages for categorical variables or as median (interquartile range) for continuous variables. Differences between groups were evaluated using the Chi-square test for categorical data and using the Mann–Whitney U test or the Kruskal–Wallis test for continuous data. 

Receiver Operating Characteristic (ROC) curve analysis was applied to examine the diagnostic performance of CAT in discriminating patients with PsO vs. patients with AD. The data are presented with areas under the curve (AUCs) and a 95% confidence interval (CI). The Youden’s Index was used to discriminate patients with PsO vs. AD [20]. The *p* level < 0.05 was set as statistically significant.

## 3. Results

Table 1 presents clinical and biochemical parameters in patients with psoriasis versus atopic dermatitis.

There was no difference in gender distribution between patients with PsO vs. patients with AD (*p* = 0.251). Patients with PsO were significantly older (*p* = 0.011) and had higher BMI (*p* < 0.001) than patients with AD. The intensity of the itch was higher in patients with AD (*p* = 0.004). There was no difference in the prevalence of smokers and disease duration between examined groups of patients. More patients with PsO used methotrexate, as compared with the corresponding AD group (25% vs. 5%, *p* = 0.013). The prevalence of comorbidities was similar in both groups, nearly 50% (*p* = 0.129). There was no difference in metabolic parameters between the examined groups. Regarding oxidative stress biomarkers, except for higher CAT activity in the AD group (*p* < 0.001), there was no difference in other biomarkers between the PsO and AD group.

The coexistence and distribution of comorbidities in patients with PsO vs. AD are presented in Table 2. The most prevalent comorbidities in both groups were hypertension, diabetes, and asthma. There was an equal percentage of patients with hypertension in both groups (35%), twice as high a percentage of patients with PsO and with diabetes as compared with the AD group (20% vs. 10%, respectively), and a higher percentage of asthma in AD group as compared to PsO group (17.5% vs. 5%, respectively).

Regarding redox homeostasis parameters, the influence of comorbidities was only shown on the activity of CAT, being higher in patients with AD with comorbidities as compared with PsO patients with comorbidities (*p* = 0.003) (data not presented).

Table 3 presents the influence of therapy on parameters of redox homeostasis in all examined patients. Significantly higher SOD activity was shown in a group of patients treated with methotrexate vs. local therapy (*p* = 0.036).

**Figure 1:**
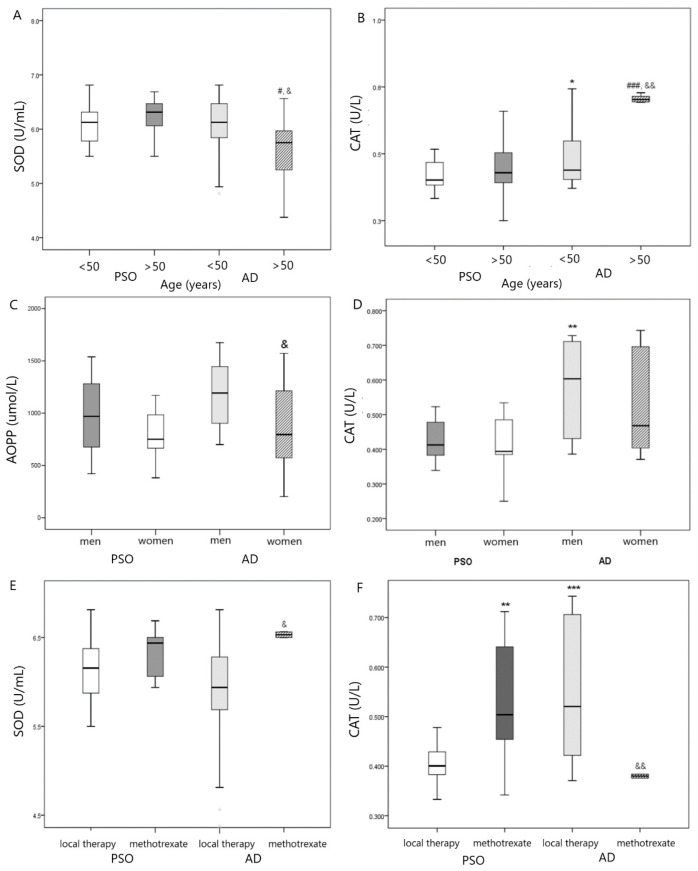
(**A**) Comparison in SOD activity in PsO vs. AD group according to age, # *p* < 0.05 vs. PsO > 50 years; & *p* < 0.05 vs. AD <50 years; (**B**) Comparison in CAT activity in PsO vs. AD group according to age, * *p* < 0.05 vs. PsO < 50 years; ### *p* < 0.001 vs. PsO > 50 years; && *p* < 0.01 vs. AD < 50 years; (**C**) Comparison in AOPP level in PsO vs. AD group according to gender, & *p* < 0.05 vs. AD men; (**D**) Comparison in CAT activity in PsO vs. AD group according to gender, ** *p* < 0.01 vs. PsO men; (**E**) Comparison in SOD activity in PsO vs. AD group according to therapy, & *p* < 0.05 vs. AD with local therapy; (**F**) Comparison in CAT activity in PsO vs. AD group according to therapy, ** *p* < 0.01, *** *p* < 0.001 vs. PsO with local therapy; && *p* < 0.01 vs. AD with local therapy.

When subdividing patients according to age, we found that older patients with AD (>50 years) had lower SOD activity as compared with the PsO group of similar age and younger patients with AD (<50 years) (Figure 1A) but higher CAT activity in comparison with PsO group (>50 years) and younger AD group (<50 years) (Figure 1B). Additionally, higher CAT activity was shown in the younger AD group (<50 years) as compared with the younger PsO group (<50 years) (Figure 1B).

When subdividing our patients according to gender and type of skin disease (PsO vs. AD), we found that AOPP levels were lower in women with AD in comparison with men (Figure 1C), whereas men with AD exhibited higher CAT activity than men in PsO group (Figure 1D). 

Higher SOD (Figure 1E) and lower CAT activity (Figure 1F) were found in patients with AD that used methotrexate in comparison with AD patients that used local therapy. On the contrary, patients with PsO who used methotrexate exhibited higher CAT activity in comparison with PsO patients who used local therapy (Figure 1F).

**Figure 2:**
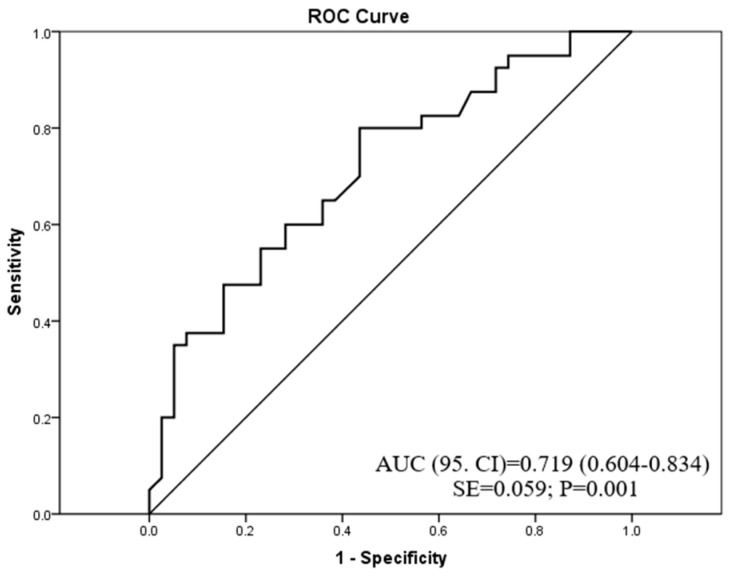
The diagnostic accuracy of catalase activity in psoriasis or atopic dermatitis prediction.

Among all examined redox homeostasis biomarkers, ROC analysis showed that only CAT exhibited good diagnostic accuracy (AUC = 0.719) in the discrimination of patients with PsO vs. patients with AD (Figure 2). A cut-off value of CAT activity was 0.436 U/L, showing that patients with CAT activity < 0.436 U/L have a high probability of belonging to the PsO group, whereas patients with CAT activity > 0.436 U/L have a high probability of belonging to the AD group.

## 4. Discussion

To our knowledge, the current study is the first to investigate several redox homeostasis parameters in patients with PsO and AD, respectively, in an attempt to make a comparative analysis between these disease entities and to find a potential biomarker that would be reliable to best discriminate patients with PsO from patients with AD.

Unlike other parameters of redox homeostasis in our study, patients with AD exhibited higher CAT activity as compared with PsO counterparts. Furthermore, ROC analysis showed that CAT had good diagnostic accuracy (AUC = 0.719) in the discrimination of patients with these disease entities.

Given the fact that there is no „ideal“ redox status biomarker that would best reflect the level of oxidative stress in chronic diseases [21], a large number of biomarkers was explored in patients with PsO and AD as separate entities in comparison with healthy controls and reported contradictory results [1,6,7,8]. We have recently shown [8] higher levels of AOPP and higher CAT activity in patients with PsO (n = 35), although no difference in MDA was found as compared with healthy subjects. Similarly, Yazici et al. [16] recorded higher AOPP levels in PsO patients as compared with healthy controls. On the contrary, Skoie et al. [14] did not find the difference in MDA and AOPP levels between examined groups.

The possible discrepancies between studies could be attributed to the coexistence of comorbidities, different disease duration, the severity of disease, medications use, food containing different levels of antioxidants that might enhance the activity/expression of the antioxidant defense system [9,21], etc.

Both PsO and AD are chronic inflammatory diseases with systemic manifestations other than skin pathology. Both diseases are related to obesity via the complex influence of proinflammatory cytokines (IL-6, IL-17, TNF-α) and adipokines, leading to cardiometabolic disturbances [4,22,23,24,25]. However, unlike clear evidence of increased prevalence of cardiometabolic diseases in patients with PsO, results concerning the link between AD and cardiometabolic risk are inconclusive [26].

Nearly half of our examined patients in both disease entities had comorbidities. Patients with PsO had higher BMI (mean values 27 kg/m^2^) as compared with BMI in AD (mean values 24 kg/m^2^). Similarly, Egeberg et al. [26] showed higher BMI in patients with PsO as compared with the general population. However, the association between AD and BMI was not so evident since they showed that a high percentage of patients with severe AD had a BMI > 30 kg/m^2^, but also a high percentage of patients with mild to moderate AD had a BMI < 25 kg/m^2^ [26].

In a comparative analysis that included patients with moderate/severe AD (n = 59), patients with PsO (n = 22), and healthy subjects (n = 18), Brunner et al. [27] demonstrated high levels of several serum atherosclerotic biomarkers exclusively in patients with AD, but not in PsO, suggesting different pathological processes in AD versus PsO. They also confirmed a positive correlation between mediators of inflammation (i.e., IL-16, PI3/elafin, E-selectin, CCL7) and AD disease severity, but not with BMI, pointing out the influence of AD on cardiometabolic risk independently of BMI [27].

Since our patients with AD had lower BMI than patients with PsO, we have further divided patients according to BMI and type of skin disease (PsO vs. AD) and found higher CAT activity in normal weight patients (BMI < 25 kg/m^2^) with AD as compared with normal weight patients with PsO. There was no difference in oxidative stress biomarkers in subgroups with BMI ≥ 25 kg/m^2^ (data not presented). This further confirms the potential influence of AD on CAT activity, independently of BMI.

In our current study, patients with PsO were older than their AD counterparts, similarly as in a previously mentioned study [27]. Therefore, we further subdivided patients according to age. In line with this, we found that older patients with AD (>50 years) had lower SOD activity as compared with the PsO group of similar age and younger patients with AD (<50 years). On the contrary, older patients with AD (>50 years) had higher CAT activity in comparison with the PsO group (>50 years) and younger AD group (<50 years). Additionally, higher CAT activity was shown in the younger AD group (<50 years) as compared with the younger PsO group (<50 years). These differences might be attributed to the influence of age on redox homeostasis, in addition to other effects of comorbidities, therapy, and smoking [9,21], but also support the assumption that the treatment for PsO and AD might be age-related [13].

Galiniak et al. [28] showed higher oxidative stress (i.e., AOPP levels) in patients with AD (n = 21) in comparison with healthy controls (n = 14) and confirmed a positive correlation between AOPP and age in these patients. 

The diagnosis of PsO and AD are more often confirmed in women than in men [25]. When subdivided our patients according to gender and type of skin disease (PsO vs. AD), we found that men with AD exhibited higher CAT activity than men in the PsO group, whereas AOPP levels were lower in women with AD in comparison with men with AD which is opposite to Chen et al. [12] who did not confirm a gender difference in urinary 8-OHdG in children with AD. The findings from our current study support the idea that the treatment for PsO and AD should be gender-related (i.e., specifically in men).

Concerning the coexistence of comorbidities, higher CAT activity was shown in the AD group with comorbidities than in PsO counterparts (data not presented). The comorbidities further enhance free radicals production, contributing to an additional burden to the already high pro-oxidant milieu. If this oxidative distress is prolonged, the previously increased antioxidants that cope with free radicals become depleted [29].

When the type of therapy was taken into account, higher SOD activity was found in patients (PsO and AD together) that used methotrexate in comparison with patients that used local therapy, which could be explained in part by a compensatory increase in antioxidant enzymes activity (i.e., SOD) in patients with severe disease as compared with patients that used only local therapy. Additionally, higher SOD and lower CAT were found in patients with AD that used methotrexate in comparison with AD patients that used local therapy. On the contrary, patients with PsO who used methotrexate exhibited higher CAT activity in comparison with PsO patients who used local therapy. These divergent results could, in part, be attributed to the influence of medications on oxidative stress levels. Methotrexate is a widely accepted therapy in PsO due to its immunosuppressive, anti-inflammatory, and antiproliferative effects [9]. However, previous studies showed that methotrexate has a contradictory effect on redox homeostasis, i.e., exhibiting proapoptotic and prooxidative properties on one side and decreasing free radicals production on the other via inhibition of signaling pathways related to inflammation [6,9]. Piskin et al. [30] showed that twelve-week treatment with methotrexate led to an increase in oxidative stress (i.e., MDA), as well as a decrease in antioxidant enzymes activity (SOD and CAT) in patients with PsO.

Among all examined oxidative stress biomarkers in our study (i.e., MDA, AOPP, SOD, and CAT), only CAT showed good discriminatory accuracy (AUC = 0.719) between AD and PsO patients with a cut-off level of 0.436 U/L. This result suggests that patients with CAT activity lower than this value have a high probability of belonging to the PsO group and vice versa; those with CAT activity higher than 0.436 U/L have a high probability of belonging to the AD group.

The conversion of the highly reactive biomolecule of hydrogen peroxide into water and oxygen is the mechanism by which CAT neutralizes the harmful effects of free radicals [18]. Although our findings point out the diagnostic significance of CAT, there are several limitations of this study that need to be mentioned. The causality between CAT and examined disease entities cannot be confirmed due to the cross-sectional design of the study. A relatively small sample size could affect the results of the study. Additionally, the inclusion of healthy controls could contribute to a more profound comparative analysis of biomarkers of oxidative stress in PsO and AD versus healthy populations. The difference in age and BMI in PsO and AD, similar to previous studies [26,27], could also affect the results of the current study. However, as our patients were consecutively included in this study, we have further made comparisons between oxidative stress biomarkers according to age, gender, BMI, comorbidities, and medication use (methotrexate vs. local therapy). We were limited to the data concerning some medications related to co-existent diseases, physical activity, and food consumption rich with antioxidants that could increase the antioxidant defense system activity [9,14,21]. Since we were limited to the histology analysis and given the fact that we have measured oxidative stress parameters in the blood (i.e., sera samples) of patients with AD and PsO, we were not able to conclude how CAT performs compared to histology analysis. Further studies are needed to extend these findings.

On the other hand, the strength of the current study lies in the fact that it is the first study that evaluated several biomarkers of oxidative stress in patients with PsO and AD. We have also provided deeper statistical analysis when dividing patients with PsO and AD subgroups according to several previously mentioned criteria (i.e., age, gender, BMI, the coexistence of comorbidities, and therapy) to obtain a clearer picture of the impact of the mentioned factors on these disease entities. 

## 5. Conclusions

The antioxidant enzyme CAT was shown to be a specific and reliable biomarker and exhibited good diagnostic accuracy in the discrimination of patients with AD from those with PsO. Age and gender-specific therapeutic antioxidant approaches (specifically in men) could be helpful in PsO and AD. The obtained results could also suggest the importance of dietary habits containing food rich in antioxidants, as well as the use of antioxidants as a potential therapeutic strategy in the treatment of the examined skin inflammatory diseases. Longitudinal studies are necessary to support our results.

## Figures and Tables

**Table 1:** Clinical and biochemical parameters in patients with psoriasis versus atopic dermatitis.

Parameter	PsO	AD	*p*
Sex			
men, n (%)	22 (55)	18 (45)	0.251
women, n (%)	18 (45)	22 (55)	
Age (years)	50 (39–67)	42 (34–51)	0.011
BMI (kg/m^2^)	27.0 (24.0–30.8)	23.8 (21.7–25.0)	<0.001
Smoking, n (%)			
No	22 (55)	23 (58)	0.500
Yes	18 (45)	17 (42)	
Disease duration (years)	8.0 (5.0–13.5)	10 (5.0–17.5)	0.284
Intensity of itch	6.0 (5.0–8.0)	8.0 (7.5–9.0)	0.004
Therapy local, n (%)	30 (75.0)	38 (95.0)	0.013
Methotrexate, n (%)	10 (25.0)	2 (5%)	
Comorbidities			
No	19 (47.5)	20 (50.0)	0.500
Yes	21 (52.5)	20 (50.0)	
Glucose (mmol/L)	5.3 (4.9–5.9)	5.2 (4.9–5.4)	0.178
TC (mmol/L)	5.02 (4.39–6.12)	4.60 (4.06–5.26)	0.227
HDL-c (mmol/L)	1.37 (1.09–1.63)	1.54 (1.22–1.63)	0.233
LDL-c (mmol/L)	2.88 (2.14–3.62)	2.47 (2.14–3.54)	0.564
TG (mmol/L)	1.68 (0.93–2.34)	1.13 (0.75–1.90)	0.114
MDA (μmol/L)	4.00 (1.67–7.49)	5.15 (2.85–8.62)	0.146
AOPP (μmol/L)	909 (676–1035)	1086 (724–1300)	0.145
SOD (U/mL)	6.19 (5.94–6.44)	6.00 (5.75–6.38)	0.162
CAT (U/L)	0.412 (0.383–0.478)	0.521 (0.420–0.706)	<0.001

**Table 2:** Coexistence of comorbidities in patients with psoriasis versus atopic dermatitis.

Comorbidity	PsO	AD	*p*
Without comorbidities, n (%)	19 (48.7)	20 (50.0)	0.261
Hypertension, n (%)	6 (15.4)	5 (12.5)	
Diabetes, n (%)	3 (7.7)	0 (0)	
Obesity, n (%)	2 (5.1)	4 (10.0)	
Asthma, n (%)	1 (2.6)	2 (5.0)	
Hypertension + asthma, n (%)	1 (2.6)	5 (12.5)	
Hypertension + diabetes, n (%)	5 (12.8)	4 (10.0)	
Hypertension + fatty liver disease, n (%)	2 (5.1)	0 (0)	
Hypertension (total *), n (%)	14 (35)	14 (35)	0.129
Diabetes (total), n (%)	8 (20)	4 (10)	
Asthma (total), n (%)	2 (5)	7 (17.5)	

* total means as a single comorbidity or in combination with other comorbidities.

**Table 3:** The influence of therapy on parameters of redox homeostasis in all examined patients.

Parameter	Local Therapy n = 68	METHOTREXATE n = 12	*p*
SOD (U/mL)	6.06 (5.75–6.34)	6.50 (6.09–6.53)	0.036
CAT (U/L)	0.43 (0.39–0.68)	0.46 (0.41–0.59)	0.848
MDA (μmol/L)	4.83 (2.60–8.64)	4.33 (1.92–5.36)	0.553
AOPP (μmol/L)	971 (681–1250)	976 (761–1064)	0.856

Since patients with AD had lower BMI than patients with PsO, we have further divided them according to BMI and type of skin disease (PsO vs. AD) and found higher CAT activity in normal weight patients (BMI < 25 kg/m^2^) with AD as compared with normal weight patients with PsO. There was no difference in oxidative stress biomarkers in subgroups with BMI ≥ 25 kg/m^2^ (data not presented).

## Data Availability

Data are available upon reasonable request (contact person: aleksandranklisic@gmail.com).
